# New Methods for Calculating LDL-Cholesterol and Related Biomarkers of Atherosclerotic Cardiovascular Disease Risk

**DOI:** 10.1007/s11883-026-01398-z

**Published:** 2026-02-25

**Authors:** Anna Wolska, Yeganeh Mansourian, Rafael Zubirán, Maureen Sampson, Alan T. Remaley

**Affiliations:** 1 Lipoprotein Metabolism Laboratory, Translational Vascular Medicine Branch, National Heart, Lung, and Blood Institute, National Institutes of Health, 9000 Rockville Pike, Bldg. 10/Rm. 8N220, Bethesda, MD 20892 USA; 2 Department of Laboratory Medicine, Clinical Center, National Institutes of Health, Bethesda, MD USA

**Keywords:** Biomarkers, Cardiovascular disease, Equations, LDL-cholesterol, Risk factors, Small dense LDL

## Abstract

**Purpose of Review:**

This review describes the recently developed equations for calculating Low-density lipoprotein cholesterol (LDL-C), and equations for estimating small dense LDL-cholesterol (sdLDL-C), and LDL-triglycerides (LDL-TG) for atherosclerotic cardiovascular disease (ASCVD) risk assessment.

**Recent Findings:**

The new Modified Sampson-NIH equation provides a more accurate estimation of LDL-C across a wide range of TG levels compared to the traditional and still commonly used Friedewald equation. Furthermore, it is more accurate compared to other equations at the low LDL-C cutpoints used for high-risk and very high-risk ASCVD patients and is valuable for deciding the need for additional lipid-lowering therapy. New equations for calculating sdLDL-C and LDL-TG use the same lipid parameters as for calculating LDL-C but offer additional insights into atherogenic lipoprotein burden.

**Summary:**

High plasma TG and very low LDL-C concentrations necessitate more accurate LDL-C calculations, which can be readily adopted without additional cost to improve ASCVD risk management.

## Introduction

Elevated low-density lipoprotein cholesterol (LDL-C) is a well-established causal risk factor in the development of atherosclerotic cardiovascular disease (ASCVD) [1]. Accordingly, US [2] and international [3, 4] guidelines have uniformly endorsed the use of LDL-C for initial risk stratification in primary prevention of ASCVD and also as a therapeutic target [5–7].

After the 1972 landmark paper by WT Friedewald and his NIH colleagues [8], the Friedewald equation quickly became the main method used for measuring LDL-C. It eliminated the need for a laborious and time-consuming β-quantification (BQ) method that depends upon the density gradient separation of lipoproteins by ultracentrifugation, followed by precipitation and measurement of cholesterol in defined density fractions [9]. Importantly, the BQ method still serves as the reference method for the standardization of all routine diagnostic assays for LDL-C. A major advantage of the Friedewald equation is that it only relies upon the results of the standard lipid panel, namely total cholesterol, triglycerides (TG) and high-density lipoprotein-cholesterol (HDL-C).

Despite its widespread utilization, the Friedewald equation was well recognized to have several important limitations, particularly in the setting of hypertriglyceridemia [10]. In addition, it is relatively inaccurate at low levels of LDL-C, which did not pose a major issue in the era when patients were largely just treated with statins and infrequently reached very low levels of LDL-C. With proprotein convertase subtilisin/kexin type 9 (PCSK-9) inhibitors plus statins enabling much lower LDL-C levels, and the growing evidence that aggressive LDL-C reduction benefits high-risk and secondary-prevention patients, accurate measurement of very low LDL-C has now become a critical clinical concern [11]. In response to the limitations of the Friedewald equation, the Martin-Hopkins equation [12] was developed in 2013 to improve LDL-C estimation across a wider range of TG levels.

Besides calculating LDL-C, some clinical laboratories use fully automated direct (homogenous) assays for LDL-C [13], which are distinct from the BQ reference method [14]. Direct homogenous assays for both LDL-C and HDL-C, which selectively measure either LDL-C or HDL-C, were first developed in the mid-1990s [13, 15, 16]. Direct (homogenous) HDL-C assays [16] were quickly adopted, because they eliminated the need for a manual LDL-C precipitation step used in the processing of the standard lipid panel. In contrast, direct LDL-C assays [13] have never been as widely utilized, because of the uncertainty on whether the extra cost in performing the assay outweighs any potential analytical advantage they may have over a free calculation. Several studies have shown that direct LDL-C assays on dyslipidemic samples can sometimes yield LDL-C results that are substantially lower or higher [17–19] than the BQ reference method.

The latest advance in calculating LDL-C was the development of the Sampson-NIH equation in 2020 [20] and its subsequent improvement in 2025 called the Modified Sampson-NIH equation [21]. Equations for estimating other aspects of LDL atherogenicity, such as small dense LDL‑cholesterol (sdLDL‑C) [22] and LDL‑triglycerides (LDL‑TG) [23] have also recently been described.

This review focuses on the different commonly used equations for estimating LDL-C and other LDL related equations. The rationale for how the various equations were developed and their strength and weaknesses for ASCVD risk assessment will be discussed. We also describe a new method called the Lipid-ratio plot [24] for how a clinical or research laboratory can indirectly examine the accuracy of their LDL-C method without ultracentrifugation.

## LDL Structure and Composition

**Fig. 1:**
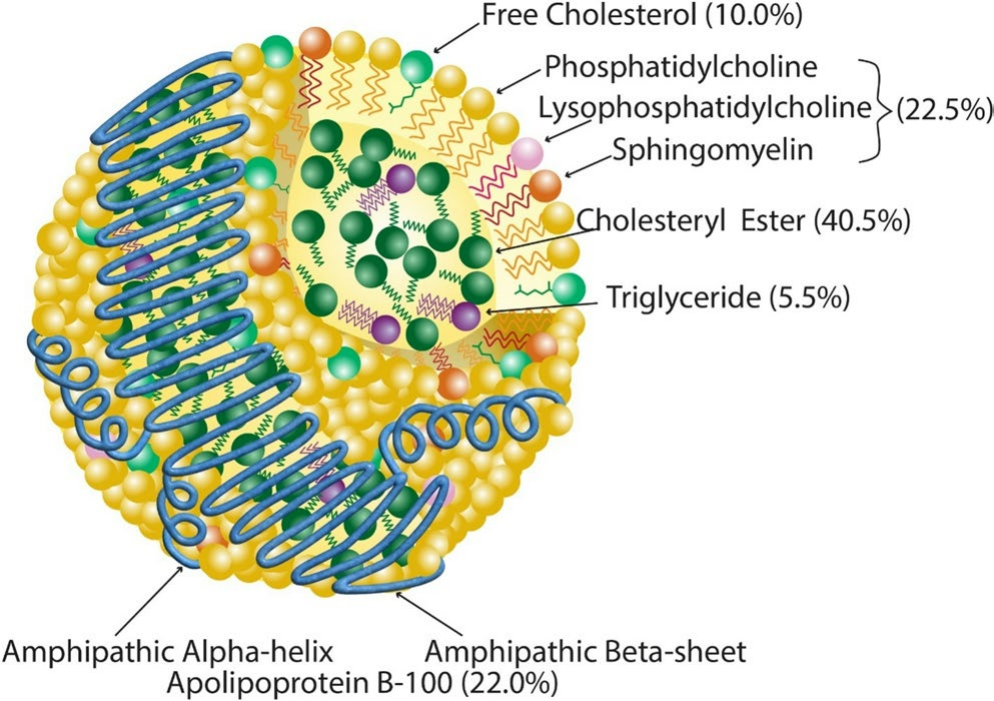
Structure and composition of LDL. LDL consists of a lipid core containing cholesteryl esters and triglycerides, surrounded by a monolayer of phospholipids and other polar lipids [29]. It contains a single molecule of apolipoprotein B-100, which forms amphipathic α-helical and β-sheet domains [28]. Adapted with permission from Wolters Kluwer Health, Inc.: Wolska A, Remaley AT. Measuring LDL-cholesterol: what is the best way to do it? Current Opinion in Cardiology. 2020;35(4):406–413 [10]. https://journals.lww.com/co-cardiology Copyright © 2020 Wolters Kluwer Health, Inc.

LDL (Fig.1) are sphere-like, heterogeneous complexes of lipids and proteins and are approximately 19–22 nm in diameter, with a density ranging between 1.019 and 1.063 g/mL [25, 26]. LDL along with other types of lipoprotein particles in the circulation are the major transporter of hydrophobic and amphipathic lipids. The core of LDL contains the most hydrophobic lipids, namely TGs and cholesteryl esters. The remaining lipids carried by LDL like free cholesterol, phospholipids, and sphingomyelin all contain a polar functional group and thus are found as a monolayer on the surface of LDL [10]. Although very low-density lipoproteins (VLDL), the precursor from which LDL particles are largely formed, contain a wide variety of proteins, the primary protein on LDL is apoB100 (hereafter referred to as apoB). It is a large protein containing 4536 amino acids and is present as a single copy per particle [27]. A recent cryo-electron microscopy structure of LDL revealed how apoB is configured around the LDL particle [28]. Approximately half of the protein sequence forms a large β-sheet structure that forms a circumferential belt around the middle of LDL. Off to the sides of this β-belt, amphipathic α-helices are present that form a cage-like structure surrounding the lipids carried by LDL. The main ligand binding site for the removal of LDL by the LDL receptor is on the β-belt region.

In normolipidemic subjects, cholesteryl esters account for the largest fraction of LDL particle mass, followed by phospholipids, apoB, free cholesterol, and TGs [29]. Historically, the quantification of LDL burden focused exclusively on LDL-C as a surrogate for atherogenic LDL mass. LDL-C includes both cholesteryl esters and free cholesterol. It is typically measured by a coupled enzymatic reaction of cholesterol after cleavage of the fatty acid from any cholesteryl esters present [30]. Emerging evidence, as will be discussed in more detail later, suggests that LDL atherogenicity may sometimes be better related to other metrics besides LDL-C. One such alternative that is increasingly being used in routine clinical practice is immunoassay testing for apoB. It provides a direct measure of all apoB-containing lipoprotein particles and is more strongly associated with ASCVD than LDL-C [31, 32].

## Commonly Used Equations for Estimating LDL-C

### Friedewald Equation

The Friedewald equation (**F-LDL-C**) [8] (Table 1), which was developed at the NIH by William T. Friedewald, Robert I. Levy and Donald S. Fredrickson, is based on the following simple premise. A fasting sample, which typically does not contain any intestinally derived chylomicrons, should only contain cholesterol transported by either LDL, VLDL or HDL. Hence, cholesterol on LDL can be calculated by simply subtracting from total plasma cholesterol, any cholesterol that is present on HDL and VLDL. Relatively simple methods were already developed at the time for measuring HDL-C, which depend upon the precipitation and removal of LDL and VLDL after the addition of a polyanion and usually a cation, such as manganese or magnesium [33]. VLDL-C is estimated, according to the Friedewald equation, as TG divided by 5 (when in mg/dL) and is consistent with a previous observation of Robert Levy [34] of an approximate 5:1 stoichiometric ratio between TGs and cholesterol in VLDL. The original Friedewald equation [8] was designed to match by linear regression a relatively small BQ dataset, but it has proved to be remarkably robust.

**Table 1:** Equations for estimating LDL-C and other LDL related lipid measures

Equation	LDL-C Formula*	Study Method	Study Population	Advantages	Disadvantages	Ref.
Friedewald: F-LDL-C	$\:\mathrm{C}-\mathrm{H}-\:\frac{\mathrm{T}}{5}$	BQ	*n* = 448; Lipid clinic	Simple, inexpensive.	Fasting samples preferred. Do not use TG > 400 mg/dL.	[8]
Martin-Hopkins: M-LDL-C	$\:\mathrm{C}-\mathrm{H}-\:\frac{\mathrm{T}}{\mathrm{F}}$	VAP	*n* = 1,350,90; General population	More accurate than F-LDL-C. Recommended by guidelines for low LDL-C <70 mg/dL.	Not based on reference method. Requires look-up table of adjustable factors (F). Do not use TG > 400 mg/dL.	[12]
Extended Martin-Hopkins: eM-LDL-C	$\:\mathrm{C}\:-\mathrm{H}-\:\frac{\mathrm{T}}{\mathrm{F}}$	VAP	Same as above and includes 111,939 patients with hypertriglyceridemia	Preferred over M-LDL-C when TG > 400 mg/dL.	Not based on reference method. Requires use of a look-up table of adjustable factors (F). Do not use TG > 800 mg/dL.	[42]
Sampson-NIH: S-LDL-C	$\:\frac{\mathrm{C}}{0.948}\:-\:\frac{\mathrm{H}}{0.971}\:$ $\:-\left(\frac{\mathrm{T}}{8.56}+\frac{\mathrm{T}\times\:\mathrm{N}}{2140}\:-\:\frac{{\mathrm{T}}^{2}}{16100}\right)-9.44$	BQ	*n* = 18,656; Lipid clinic	Improved accuracy for a broad TG range.	Study population had a limited number of patients with LDL-C < 70 mg/dL. Do not use TG > 800 mg/dL. Patients with Type III dysbetalipoproteinemia were excluded.	[20]
Enhanced Sampson-NIH: eS-LDL-C	$\:\frac{\mathrm{C}}{1.15}\:-\:\frac{\mathrm{H}}{1.25}\:-\:\frac{\mathrm{T}}{6.99}\:$ $\:-\:\frac{\mathrm{T}\times\:\mathrm{N}}{1120}+\frac{{\mathrm{T}}^{2}}{8910}+\:\frac{\mathrm{T}\times\:\mathrm{B}}{1240}+\frac{\mathrm{B}}{4.54}-4.73$	BQ	*n* = 24,406; General population	Improved accuracy for VLDL-C and LDL-C.	Requires apoB. Do not use TG > 800 mg/dL.	[45]
Modified Sampson-NIH: mS-LDL-C	$\:\mathrm{N}\:-\:\frac{\mathrm{T}}{8.37}\:-\:\frac{\mathrm{T}\times\:\mathrm{N}}{2640}$ $\:+\frac{{\mathrm{T}}^{2}}{17400}$	BQ	*n* = 34,023; General population and FOURIER trial	Preferred when LDL-C < 70 mg/dL. Higher TG limit than other equations.	Optimized mainly for low LDL-C, high-risk populations. Do not use TG > 1000 mg/dL. Patients with Type III dysbetalipoproteinemia were excluded.	[21]
Estimated sdLDL-C: e-sdLDL-C	$1.\;lb\mathrm {LDLC}=1.43\times\mathrm{LDLC}$ $-\left(0.14\times\left(\ln\left(\mathrm T\right)\times\mathrm{LDLC} \right)\right)-8.99$ $2.\;sd\mathrm{LDLC}=\mathrm{LDLC}-\;lb\mathrm {LDLC}$	direct sdLDL-C	*n* = 20,171; Lipid clinic	Advanced lipid testing not needed.	Unclear clinical utility.	[22]
Estimated LDL-TG: e-LDL-TG	$\:\frac{\mathrm{T}}{38.5}+\:\frac{\mathrm{N}}{5.75}+\frac{9.75\:\mathrm{T}}{\mathrm{N}}$ $\:+\:\frac{244}{\mathrm{H}}\:-2.95\:$	BQ	*n* = 40,202; General population	Advanced lipid testing not needed.	Unclear clinical utility.	[23]

*T:* Triglycerides, *C:* Total Cholesterol, *H:* HDL-C, *B:* apoB, *N:* Non-HDL-C, *F:* Adjustable factor, *lb:* large buoyant, *sd:* small dence

It was known from the outset that the TG/5 estimate of VLDL-C becomes increasingly unreliable as TG levels rise. This is the reason for the widespread practice of not calculating LDL-C by the Friedewald equation when TG > 400 mg/dL [8, 12]. In hypertriglyceridemic samples there is an increase in large TG-rich VLDL, which leads to an underestimation of LDL-C [20, 35]. In contrast, patients with Familial Dysbetalipoproteinemia have an enrichment of cholesterol on VLDL, and the calculation leads to an overestimation of LDL-C in these patients [36]. This limitation of the Friedewald equation became even more apparent in recent times given the growing prevalence of hypertriglyceridemia and the shift towards non-fasting lipid panels for cardiovascular risk assessment. Low LDL-C samples are also a challenge, because VLDL-C represents a greater fraction of total cholesterol and hence errors in the estimate of VLDL-C have a proportionally a greater effect on LDL-C accuracy. We previously found that as many as a third of all individuals with an LDL-C < 70 mg/dL by the Friedewald equation are, in fact, falsely low by as much as 30 mg/dL (0.77 mmol/L) [37]. This can lead to an undertreatment of high-risk patients and can make it difficult to get reimbursement for the new potent but also more expensive lipid-lowering therapies like PCSK9-inhibitors.

### Martin-Hopkins Equation

Although many different LDL-C equations have been proposed over the years [38], the first major improvement over the Friedewald equation was the Martin-Hopkins equation (**M-LDL-C**) in 2013 [12]. It is almost identical to the Friedewald equation (Table 1), except it uses a variable denominator for dividing TG to obtain VLDL-C, which can be found in a 180-cell table. It is more accurate, because it was found that the optimum denominator for estimating VLDL-C depends not only on TG but also nonHDL-C. At higher TG levels, the optimal denominator becomes greater, whereas for nonHDL-C, the optimal denominator becomes lower as nonHDL-C increases. This relationship was empirically derived from over a million patients that were analyzed by a commercial laboratory, using the Vertical Autoprofile (VAP) procedure [39]. It is a rapid ultracentrifugation method for the separation of lipoproteins, followed by the enzymatic measurement of cholesterol. The VAP method to which the Martin-Hopkins equation was trained against can sometimes vary from the BQ reference method. This is most likely due to its use of a vertical‑tube rotor, rather than the fixed‑angle rotor used in the BQ method. The VAP method appears to under-recover VLDL‑C in high‑TG samples [40], likely because VLDL adheres to the sides of the tube [41]. This results in an overestimation of LDL‑C by VAP and consequently an overestimation in also the Martin-Hopkins equation. Although the bias is smaller, VAP also yields low VLDL‑C and falsely high LDL‑C in low‑TG samples [20, 40] when compared to the BQ reference method.

Because of its improved accuracy, the 2018-AHA/ACC/Multi-Society Cholesterol Guideline assigned a Class IIa recommendation to use the Martin-Hopkins equation for a ‘modified estimation’ of LDL-C in patients with LDL-C < 70 mg/dL and TG > 150 mg/dL [2]. Originally, the equation was only recommended when TG < 400 mg/dL, but a modified table of denominators was later developed so that the extended Martin–Hopkins equation (**eM-LDL-C**) can be used for TG levels up to 800 mg/dL [42].

### Sampson-NIH Equation

In 2020, a new estimation method called the Sampson-NIH equation (**S-LDL-C**), which is suitable for TG up to 800 mg/dL, was described [20]. It is in the form of a bivariate quadratic equation (Table 1) and like the other equations depends only upon the standard lipid panel. The higher order mathematical terms of this equation better accounts for the complex surface-volume relationships that determine the TG content in the core of lipoproteins. Like the Friedewald equation, it was developed by least-square regression to match the BQ reference method. The Sampson-NIH equation was reported to be the most accurate equation for estimating LDL‑C, especially at very high levels of TGs, resulting in less misclassification at various thresholds throughout the LDL-C range [37].

The continuous nature of the Sampson-NIH equation is another advantage in that it avoids the discontinuous “jumps” in calculated VLDL-C that sometimes occurs with the Martin-Hopkins equation due to the quantile nature of its factor table, particularly at higher TG levels when the factor is determined from a relatively wide TG interval [43]. The use of a large number of discrete factors in the Martin–Hopkins equation also makes it more difficult to implement. For most clinical laboratory information systems, a continuous equation can be directly entered into the system by the user, whereas an equation that depends upon a look-up table of factors may require additional information technology support. A limitation of the Sampson-NIH equation is that the original BQ training dataset had only a limited number of samples with LDL-C below 70 mg/dL, thus limiting its accuracy below this point [20].

### Enhanced Sampson-NIH Equations

A modification of the Sampson-NIH equation for first measuring VLDL-C [44] and later also LDL-C [45] called the Enhanced Sampson-NIH equation (**eS-LDL-C**) has been reported. Unlike other LDL-C equations, it includes apoB as an independent variable (Table 1). ApoB provides additional information related to total particle number of apoB-containing lipoproteins, which includes both LDL and VLDL. This information cannot be ascertained from the standard lipid panel and as a consequence, the Enhanced Sampson-NIH equation shows a closer correspondence to the BQ reference method than other equations [44]. The clinical utility of this LDL-C equation is somewhat limited, because apoB by itself has been shown in numerous studies to be superior to LDL-C as an ASCVD biomarker [31, 32].

One potential application of Enhanced Sampson-NIH equation, but requires additional studies, is in the estimation of remnant cholesterol, which is essentially VLDL-C. Given the strong association of remnant cholesterol with ASCVD [46], a more accurate partitioning of cholesterol on nonHDL between remnants and LDL could improve the diagnostic performance or remnant cholesterol. In most previous studies, remnant cholesterol is simply calculated as nonHDL-C minus LDL-C, as determined by the Friedewald equation. This approach ranks remnant cholesterol the same as TG, and thus does not provide any better discrimination for ASCVD risk than plasma TG [47].

Another potential application for this equation is for the detection of dysbetalipoproteinemia [40]. In this type of dyslipidemia there is an enrichment of cholesterol on VLDL due to delayed plasma clearance, and it can occur in a wide variety of conditions, such as obesity and diabetes, as well as in patients who are homozygous for the apoE2 allele and have Familial Dysbetalipoproteinemia [26]. However, VLDL-C or the VLDL-C/TG ratio as calculated by this equation is not as specific for diagnosing Familial Dysbetalipoproteinemia as the nonHDL-C/apoB ratio [48], because it reflects all causes of dysbetalipoproteinemia.

### Modified Sampson-NIH Equation

The latest equation for estimating LDL-C is the Modified Sampson-NIH equation (**mS-LDL-C**), which was reported in 2025 [21] to be particularly well suited for patients with low LDL-C. It is simpler than the original Sampson-NIH equation, because the two terms for total cholesterol and HDL-C were combined into nonHDL-C with a coefficient of one (Table 1). It also does not contain an intercept like the original equation.

Recent guidance from the American College of Cardiology [49] now recommends considering adding ezetimibe and/or PCSK9-inhibitor therapy on top of statin therapy when LDL-C remains ≥ 55 mg/dL in very-high-risk patients. Similarly, the European Society of Cardiology/European Atherosclerosis Society [50] advises an LDL-C target goal < 55 mg/dL for ASCVD patients, with an even lower target of < 40 mg/dL for those with recurrent ASCVD events. To address the need for more accurate measurement of very low LDL-C levels, the Modified Sampson-NIH equation was developed, using a more contemporary BQ dataset containing patients on the latest lipid lowering therapies. The training dataset also included 9605 lipid test results from the FOURIER clinical trial of the PCSK9-inhibitor Evolocumab [51], which in some cases lowered LDL-C below 10 mg/dL. In a separate validation dataset, it showed the best concordance at both the 55 mg/dL and 70 mg/dL cutpoints compared to other LDL-C equations. Although more studies are needed, a recent follow-up study found that the Modified Sampson-NIH equation was not only superior to other commonly used LDL-C equations but also to three different direct LDL-C assays at the 55 mg/dL and 70 mg/dL cutpoints [19].

### The Lipid-Ratio Plot

Since the publication of the Martin-Hopkins and the other new LDL-C equations, there have been a large number of reports comparing their accuracy [43, 52]. In the majority of these reports, however, the main comparison method was a direct LDL-C assay or sometimes another calculation method. This is understandable given the inability of most clinical laboratories to perform the BQ reference method, but it raises an issue about the validity of such studies, because of the known biases in direct and calculation methods [17–19, 38, 53]. It has also been discovered that the input of the lipid values used for any equation can affect its overall accuracy [54]. Although the tests that make up the standard lipid panel are relatively robust and accurate [55], a bias in these assays can help mitigate or make worse any inherent bias in an equation for calculating LDL-C.

To address these concerns, a Lipid-ratio plot [24] was developed to indirectly compare either a calculated or measured LDL-C to the BQ reference method. It depends upon the observation that the ratio of LDL-C to nonHDL-C is inversely related to the square root of TG divided by nonHDL-C. The slope and intercept of this relationship for any LDL-C method is compared against what was observed in two large BQ databases. By simulation analysis, it was observed that a minimum of 80 samples is needed to confidently determine the slope and intercept on the Lipid-ratio plot for any LDL-C method.

**Fig. 2:**
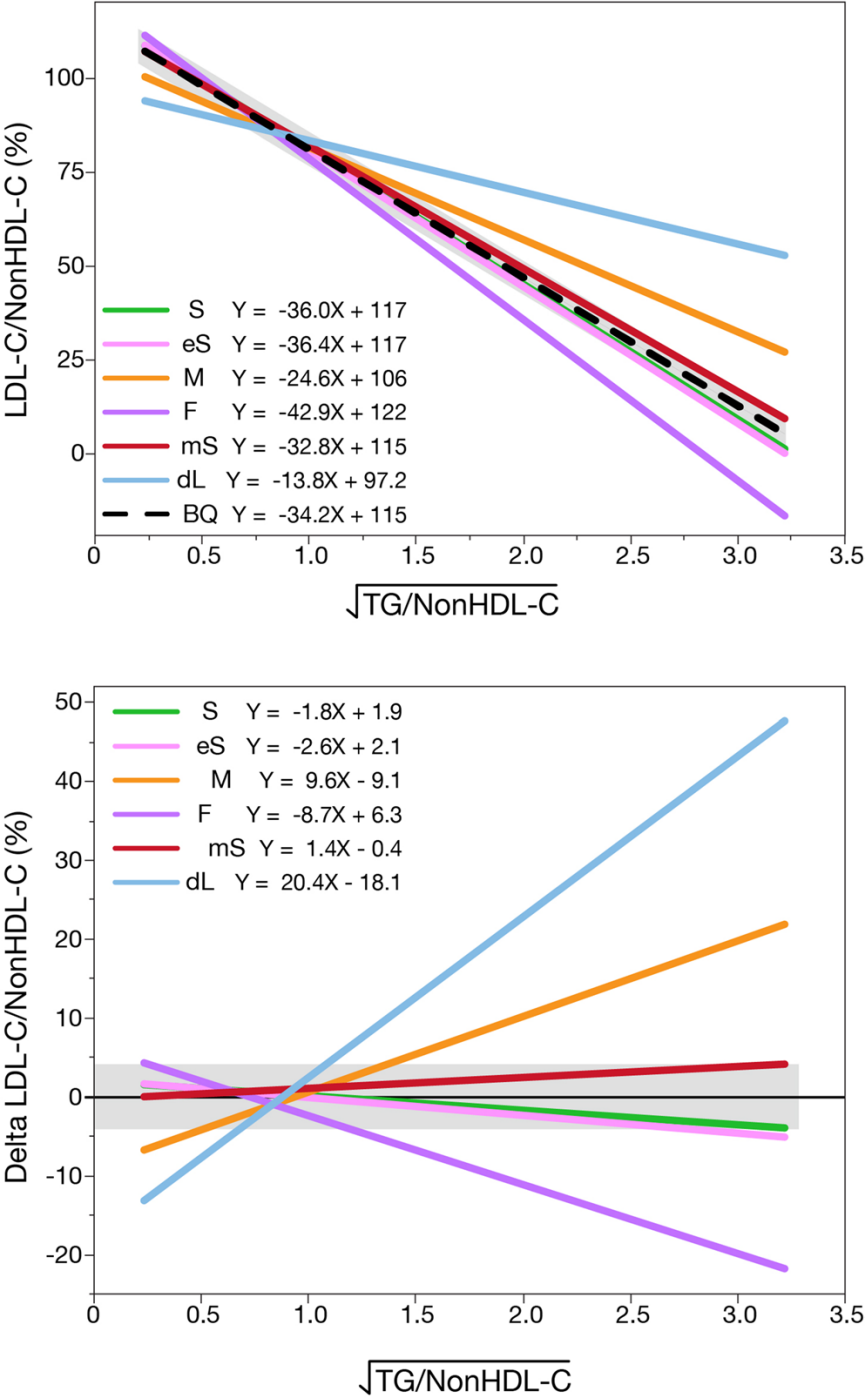
Lipid-ratio plot for assessing the accuracy of LDL-C methods. The top panel shows LDL-C/NonHDL-C (%) plotted against the square root of TG/NonHDL-C, illustrating how each method performs across increasing TG/NonHDL-C levels. The original Sampson-NIH (S, green), enhanced Sampson-NIH (eS, pink) and modified Sampson-NIH (mS, red) equations align most closely with the β-quantification line (BQ, dashed black), indicating minimal bias. The Martin-Hopkins (M, orange) equation shows a moderate bias, while the Friedewald (F, purple) equation and direct LDL-C (dL, light blue) methods exhibit substantial biases. The bottom panel presents the corresponding delta plot (calculated or measured LDL-C minus BQ), better highlighting method-specific bias. Grey zone indicates the 4% acceptable bias limit for LDL-C

An example of the Lipid-ratio plot is shown in Fig. 2. In the top panel, it was found that the original Sampson-NIH, Enhanced Sampson-NIH and Modified Sampson-NIH equation all yielded results that closely align with the BQ reference method. They all showed a minimal bias across a wide range of TG levels. By contrast, the Friedewald equation, the Martin-Hopkins equation, and the one direct LDL-C assay showed results that deviated substantially from the BQ reference method, particularly at high TG/nonHDL-C ratios. The bottom panel is a difference plot and presents the delta LDL-C/nonHDL-C, making it easier to see any biases. The horizontal grey bar indicates the maximally recommended total bias limit of 4% for LDL-C, showing how the original Sampson-NIH equation and its two modifications all fall within this error limit, whereas the other equations and the direct LDL-C assay sometimes exceed this error limit.

## Other LDL Related Equations

### SdLDL-C Equation

Small dense LDL (sdLDL) has long been associated with the “atherogenic phenotype,” a metabolic profile frequently seen in patients with hypertriglyceridemia, diabetes, and metabolic syndrome [56]. Cholesteryl-ester transfer protein (CETP) enriches LDL particles with TGs during hypertriglyceridemia [57]. Subsequent lipolysis of TG on LDL generates smaller, denser LDL particles [58], which often coexist with low HDL-C and small HDL subclasses. These sdLDL particles have been proposed to be causally linked to ASCVD because of their greater arterial wall penetration, longer plasma half-life and increased susceptibility to oxidation [59, 60], but they may also just be a marker of an underlying metabolic profile that predisposes to atherosclerosis.

Historically, sdLDL-C could only be quantified by advanced lipid testing either involving ultracentrifugation or by electrophoresis, but a fully automated assay for sdLDL-C [61] has been developed and approved by the FDA. This has enabled a broader epidemiologic evaluation of sdLDL-C as an ASCVD risk marker, and it has been found in several large cohorts to outperform LDL-C [53].

An equation for estimating sdLDL-C, referred to as the estimated sdLDL-C equation (**e-sdLDL-C)** (Table 1), based on lipid panel test results has been described [22]. Although estimated sdLDL-C only shows a modest correlation with the direct assay for sdLDL-C, it does not require any additional testing. It could potentially be used to screen for patients who might benefit from having the direct sdLDL-C test performed. In a large UK Biobank cohort, e-sdLDL-C better predicted all-cause ASCVD than LDL-C and remained significantly associated with risk even after adjustment for apoB, suggesting that it may enhance risk stratification beyond traditional lipid markers; however, further validation is needed [62]. Estimated sdLDL-C was also reported to have a stronger association than LDL-C with high-risk features of coronary atherosclerotic plaques in psoriasis patients [63]. It demonstrated a strong correlation with measured sdLDL-C levels in nondiabetic subjects without medication, as well as in subjects with diabetes mellitus [64]. Recently, machine‑learning approaches have also been used to develop and equation for sdLDL-C and was found with the addition of other variables to achieve better correlation with the direct sdLDL-C assay [64]. Two other equations based on just the lipid panel test results for sdLDL-C have also been reported [65, 66]. More studies are needed, however, to compare these different equations and to better understand how to utilize calculated sdLDL-C in improving ASCVD risk assessment.

### LDL-TG Equation

Although most plasma TGs circulate in TG-rich lipoproteins (TRL), such us chylomicrons and VLDL, up to 20% of plasma TG can reside on LDL [67, 68] due to incomplete lipolysis or as a consequence of CETP-mediated lipid exchange [69]. When TRL clearance is impaired, TG-enriched LDL accumulates, although some of these large LDL particles undergo lipolysis and are converted to sdLDL via hepatic lipase [70].

LDL-TG was first reported as a potential ASCVD marker in 2004, when März et al. [71] showed a stronger associations of LDL-TG, as measured by the BQ method, with ASCVD and chronic inflammation than LDL-C. More recently, using a direct assay for LDL-TG not yet approved by the FDA [72], LDL-TG was identified as an independent predictor of ASCVD events and superior to LDL-C in the ARIC study [73]. In several subsequent large European cohorts and by a meta-analyses it was also confirmed to have a strong association with ASCVD risk [74]. Bayesian network analysis from the GLOBAL study further suggested a potential direct role of LDL-TG in atherosclerosis development [75].

These finding motivated the development of an equation to estimate LDL-TG, termed **e-LDL-TG** [23] (Table 1), again only using the standard lipid panel as the input. Although e-LDL‑TG correlates only modestly with directly measured LDL‑TG, it showed stronger associations with ASCVD than estimated LDL‑C in two large cohorts. In the UK Biobank, e-LDL‑TG even outperformed measured apoB and estimated sdLDL‑C as a risk marker, identifying high-risk individuals not detected by conventional lipid tests. These findings suggest that e-LDL‑TG could complement LDL‑C in initial risk assessment or serve as a risk-enhancer test. Much of its association with ASCVD, however, appears mediated by other high-risk metabolic conditions, which limits its predictive value in fully adjusted 10-year risk models.

## Conclusions

The evolution of LDL-related estimation methods reflects a growing need for better markers of ASCVD risk and is also a consequence of a broader shift in preventive cardiology from population-level approximation toward patient-specific precision. As the new landscape of lipid-lowering therapies now make possible the reduction of LDL-C into ranges that were previously impossible in clinical practice, small analytical errors in LDL-C have a greater impact on clinical care. This has led to a reassessment of long-standing methods like the Friedewald equation and the development of new approaches that are better suited for contemporary therapeutic needs.

The Modified Sampson-NIH equation nicely illustrates how mathematical modeling benchmarked to the BQ ultracentrifugation reference method can materially reduce systematic error. Most importantly, it improves accuracy within the low LDL-C range where therapeutic decisions are often the most sensitive to errors around the dichotomous thresholds used in clinical practice. Yet, analytical improvement alone does not redefine clinical risk. The long-standing dissociation between LDL-C, apoB, and particle composition remains biologically relevant, even when LDL-C estimation is optimized. Chromatographic approaches such as size-exclusion HPLC can directly resolve cholesterol across major lipoprotein fractions and reveal compositional abnormalities [76]. The complexity and cost of the method, however, currently limits their use to specialized settings rather than routine clinical testing. This highlights the need for new biomarkers that improve primary-prevention risk prediction and better capture residual risk. The future likely lies in moving beyond cholesterol-based metrics into an assessment of particle number, such as apoB, as a unifying risk biomarker, particularly for making treatment decisions [77–81].

Equation-based estimates of sdLDL-C and LDL-TG have only recently been reported, and substantial work remains to determine how best to integrate these calculations with LDL-C and other risk factors in ASCVD risk assessment. Like the situation with direct LDL-C assays, it will have to be determined in what clinical situations a calculation of these parameters is sufficient versus performing a direct assay. It is also not known whether these other LDL-related parameters, including other features that are not measured by conventional LDL-C assessment, such as electronegative LDL [82], are causally linked to the development of ASCVD or merely associated with it, which has important implications for future drug development.

In summary, recent progress in new methods for calculating cholesterol on LDL and other LDL-related biomarkers have both important practical consequences and open new areas of investigation. Modern equations improve the reliability of LDL-C testing and should replace the Friedewald equation given that its negative bias leads to the undertreatment of patients. A challenge in the future will be how to best integrate these new measures of lipoprotein particle number, lipid composition, and TRL burden onto our existing framework for meaningfully improving clinical decision making and cardiovascular outcomes.

## Key References

- Meeusen JW, Yi X, Cotten SW, Nielsen JB, Donato LJ, Jones PM, et al. Modern Low-Density Lipoprotein Cholesterol Formulas Outperform Direct Methods in Patients with Hypertriglyceridemia and Low Levels of Low-Density Lipoprotein Cholesterol. Clin Chem. 2025;71(11):1138-46.
  - Comparing three direct LDL-C assays against the reference method, beta-quantification, showcasing the high correlation of Sampson equation.
- Martins J, Rossouw HM, Pillay TS. How should low-density lipoprotein cholesterol be calculated in 2022? Curr Opin Lipidol. 2022;33(4):237-56.
  - Provides a comprehensive review of LDL-C calculation methods and offers practical guidance on choosing equations for different clinical contexts.
- Heidemann BE, Koopal C, Roeters van Lennep JE, Stroes ES, Riksen NP, Mulder MT, et al. Low-density lipoprotein cholesterol and non-high-density lipoprotein cholesterol measurement in Familial Dysbetalipoproteinemia. Clin Chim Acta. 2023;539:114-21.
  - Evaluates LDL-C and nonHDL-C in familial dysbetalipoproteinemia using equations, direct assay and gel running against ultracentrifugation and highlights limitations of standard lipid measurements in this disorder.
- Sniderman AD, Navar AM, Thanassoulis G. Apolipoprotein B vs Low-Density Lipoprotein Cholesterol and Non-High-Density Lipoprotein Cholesterol as the Primary Measure of Apolipoprotein B Lipoprotein-Related Risk: The Debate Is Over. JAMA Cardiology. 2022;7(3):257-8.
  - Argues that ApoB is the most accurate single measure of atherogenic risk and should be preferred over LDL-C or nonHDL-C for risk assessment.
- Gcingca T, Sampson M, Zubiran R, Wolska A, Meeusen J, Donato L, et al. Lipid Ratio Plot: A Simple Graphical Approach for Investigating the Accuracy of LDL Cholesterol Equations or Direct Assays. J Appl Lab Med. 2025;10(5):1154-67.
  - Proposes the lipid ratio plot as an indirect graphical method to assess accuracy of LDL-C calculation when comparing measurements against the reference method of beta-quantification.
- Johannesen CDL, Langsted A, Nordestgaard BG, Mortensen MB. Excess Apolipoprotein B and Cardiovascular Risk in Women and Men. J Am Coll Cardiol. 2024;83(23):2262-73.
  - Demonstrates that excess apoB relative to LDL-C is strongly associated with cardiovascular events in both women and men.
- Sampson M, Wolska A, Cole J, Zubirán R, Otvos JD, Meeusen JW, et al. Accuracy and Clinical Impact of Estimating Low-Density Lipoprotein-Cholesterol at High and Low Levels by Different Equations. Biomedicines. 2022;10(12):3156.
  - Compares multiple LDL-C equations across a broad concentration range of TG and models the potential clinical impact of equation choice on treatment decisions.
- Zubiran R, Sampson M, Wolska A, Remaley AT. Estimated Small, Dense LDL Cholesterol and Atherosclerotic Cardiovascular Risk in the UK Biobank. Arterioscler Thromb Vasc Biol. 2025;45(10):e512-e22.
  - Demonstrates that estimated sdLDL-C is strongly associated with incident ASCVD in comparison to traditional risk factors.

## Data Availability

No datasets were generated or analysed during the current study.
